# Hippocampal administration of chondroitinase ABC increases plaque-adjacent synaptic marker and diminishes amyloid burden in aged APPswe/PS1dE9 mice

**DOI:** 10.1186/s40478-015-0233-z

**Published:** 2015-09-04

**Authors:** Matthew D. Howell, Lauren A. Bailey, Michael A. Cozart, Brenda M. Gannon, Paul E. Gottschall

**Affiliations:** Department of Pharmacology and Toxicology, University of Arkansas for Medical Sciences, 4301 West Markham Street, Slot 611, Little Rock, AR 72205 USA; Department of Biomedical Sciences, Iowa State University, 2069 Veterinary Medicine, Ames, IA 50011 USA

## Abstract

**Introduction:**

Substantial data has shown that the lectican group of chondroitin sulfate proteoglycans are involved in inhibition of axonal plasticity in response to injury in the central nervous system. Increasing evidence indicates that lecticans may also play a role in synaptic plasticity related to memory, especially associated with aging. A recent study has shown that lectican expression is elevated at a young age in the APPswe/PS1dE9 mouse model and Alzheimer’s disease (AD) and hippocampal treatment with chondroitinase ABC reversed a loss of contextual fear memory and restored long-term potentiation. The purpose of this study was to examine the presence of a synaptic lectican in AD tissue, determine if amyloid-β (Aβ) binds to lecticans purified from brain tissue, and examine how treatment of the same AD model with chondroitinase ABC would influence plaque burden and the density of the synaptic marker synaptophysin around plaques.

**Results:**

In human superior frontal gyrus, levels of the brain-specific lectican, brevican, were significantly elevated in AD compared to non-cognitively impaired subjects, with a trend toward an increase in tissue from subjects with mild cognitive impairment. *In vitro* immunoprecipitation studies showed that brevican binds to oligomeric and fibrillar Aβ1-42, and less so to monomeric Aβ1-42. Intrahippocampal injection of 15 months APPswe/PS1dE9 mice with chondroitinase ABC resulted in a reduction of Aβ burden in the stratum lacunosum moleculare and a reversal of the loss of synaptic density surrounding plaques in the same region.

**Conclusions:**

It is possible that lecticans, particularly brevican, inhibit synaptic plasticity in this model of AD. Since the hippocampus undergoes changes in synaptic plasticity early in the disease process, it could be possible that removal of lecticans or inhibition of their signaling pathways could prolong plasticity in patients early in the disease process, and delay cognitive decline of AD progression.

**Electronic supplementary material:**

The online version of this article (doi:10.1186/s40478-015-0233-z) contains supplementary material, which is available to authorized users.

## Introduction

Numerous molecular events occur in the post-synaptic density of an excitatory synapse in response to a train of pre-synaptic action potentials. These include a change in transmission sensitivity and alterations in the structure of the dendritic spine, and these interrelated functions are essential for the formation and other aspects of memory [1–3]. While activation of NMDA receptors and increases in Ca^++^ in the post-synaptic density are key components of this process [4], additional factors contribute. One of these factors is the presence of peri-synaptic extracellular matrix (ECM), a lattice of chondroitin sulfate (CS)-bearing proteoglycans (PGs), termed lecticans (aggrecan, brevican, neurocan, and versican), bound to hyaluronic acid near their N-terminus and tenascin-R near their C-terminus [5]. Brevican is a lectican expressed at the synapse as it was originally discovered as a synapse-associated protein [6]. Lecticans, as part of this lattice are thought to provide stability to synapses, and inhibit functional and structural synaptic plasticity including the growth of dendritic spines and reversal of evoked field post-synaptic potentials. The majority of data that supports the role of lecticans in plasticity is derived from studies that injected the bacterial enzyme chondroitinase ABC (ChABC) into the brain. ChABC treatment results in removal of CS chains from lectican core proteins (and other CS-bearing PGs). These studies demonstrated that removal of CS chains stimulated plasticity as measured by functional outcomes including visual ocular dominance [7, 8], fear memory [9] and other forms of learning [10]. These findings suggest that the ECM lattice present at the synapse could be a therapeutic target for neurological disorders with diminished synaptic plasticity.

Alzheimer’s disease (AD) is the most common cause of dementia, and the earliest functional deficit most often associated with the disease is a loss of memory [11]. Synaptic alterations also occur early in AD progression; synaptic loss is observed prior to neuronal cell death, and the loss of synapses in the outer molecular layer of the hippocampal dentate gyrus is more highly correlated with cognitive impairment than other classical AD pathology including neurofibrillary tangles and senile plaques [12–15]. However, some data supports a role for both tau in neurofibrillary tangles, and Aβ surrounding plaques, as contributors to modifications of synapses in AD [16–18]. Complete understanding of these synaptic abnormalities, and the associated mechanism(s), could allow for the development of potential therapeutics that would delay, prevent or reverse synaptic changes in the disease process. Lectican-containing ECM around the synapse may function to inhibit synaptic plasticity. Further, the elevation of lecticans in AD and aging suggests this matrix has the potential to contribute to diminished synaptic plasticity [19, 20]. Interestingly, a recent study demonstrated that injection of ChABC in young AD model mice identified to have elevated lectican levels resulted in a reversal of contextual fear memory deficits and a restoration to normal long term potentiation [21].

Given these previous reports, this study aimed to examine whether intra-hippocampal administration of ChABC could ameliorate the loss of synaptic density surrounding Aβ-containing plaques in the hippocampus. For these experiments we utilized the APPswe/PS1dE9 model, a mouse shown to have substantial synaptic deficits with age [22]. While previous evidence demonstrated that CS-bearing PGs (derived from cartilage) can interact with and bind to Aβ, we also determined whether Aβ binds to PGs purified from mouse brains, and how this interaction might affect the deposition of Aβ into senile plaques in this model.

## Materials and Methods

### Human tissues and western blot

Superior frontal cortical tissue was obtained from the Rush Alzheimer’s Center, Rush Medical Center (Chicago, IL) from subjects with no cognitive impairment (NCI, n = 14, mean age 83.0 years, post-mortem interval 10.2 h), subjects with mild cognitive impairment (MCI, n = 10, mean age 88.4 years, post-mortem interval, 7.1 h), and AD subjects (n = 14, mean age 90.1 years, post-mortem interval 8.8 h). Distribution of age and gender was comparable among the three groups and neuropathological assessment was based on Braak score, CERAD diagnosis and NIA/Reagan Diagnosis (for details, see Additional file 1: Table S1). Frozen tissue was extracted in RIPA buffer (50 mM Tris, 150 mM NaCl, 2 mM EDTA, 1 % Triton-X-100, 1 % sodium deoxycholate, 0.1 % SDS, pH 7.4; all components from Sigma Aldrich, Saint Louis, MO) with protease inhibitor cocktail III (Calbiochem, LaJolla, CA). Western blots were conducted as previously described [23]. Briefly, sample extracts were diluted with 2x Laemmli buffer, heated at 95 C for 4 min and loaded onto 4-20 % gradient SDS-PAGE gels (Life Technologies, Carlsbad, CA), electrophoresed, transferred to PVDF membrane (Immobilon, EMD Millipore, Billerica, MA), and probed consecutively using standard antibody techniques. The primary antibodies used were goat anti-brevican (1:500, Santa Cruz Biotechnology sc-20555, Dallas, TX) and monoclonal rabbit anti-GAPDH (1:5000; Cell Signaling 5174, Beverly, MA). The blots were then incubated with donkey anti-goat IgG and goat anti-rabbit IgG conjugated to horseradish peroxidase (HRP, 1:20,000; EMD Millipore), the antigens visualized with Immobilon Western Chemiluminescent HRP substrate (EMD Millipore), and the blots were exposed to autoradiographic film (Denville Scientific, Metuchen, NJ). The film was scanned with an Epson Perfection V700 photo scanner (Epson, Long Beach, CA). Density of the bands were quantified using Image Studio software 4.0 (Li-Cor, Lincoln, NE). The density of the samples were divided to make a proportion of the internal control sample that was included on each gel. Then these were converted to a ratio of the GAPDH density (also as a measure of the internal control) from the same sample.

### Preparation of monomeric, oligomeric and fibrillar Aβ1-42

Synthetic Aβ1-42 and Aβ1-16 was purchased (American Peptide Company, Sunnyvale, CA), solubilized with 1 mM hexafluoro-2-propanol (HFIP; Sigma Aldrich), aliquoted into separate tubes and allowed to evaporate under a chemical hood followed by speedvac to ensure complete removal of solution, and subsequently stored at −20 C. To obtain monomeric Aβ1-42 and Aβ1-16, the peptide film was diluted to 1 mM with dimethylsulfoxide (DMSO) and subjected to brief bath sonication. The solution was then stored at −20 C. For oligomeric Aβ1-42, the peptide solubilized in 1 mM DMSO was diluted to 100 μM with ice cold phosphate-buffered saline (PBS) and placed at 4 C for 18 h or 42 h and immediately used. For fibrillar Aβ1-42, peptide solubilized in 1 mM DMSO was diluted to 100 μM with PBS, incubated in a 37 C water bath for 48 h, and then stored at −20 C for future use.

### Aβ binding assays, DEAE purification of brain tissue, immunoprecipitation

Soluble extracts were prepared from whole wild type C57Bl/6 mouse brain and purified on a diethylaminoethyl (DEAE) Fast Flow column (GE Healthcare Life Sciences, Pittsburgh, PA) with sequential elution with increasing concentrations of NaCl as described [24, 25]. This method generates an extract enriched with CS-containing proteins. This extract was used for a solid-phase binding assay to determine potential binding with Aβ1-42 and Aβ1-16 using modification of a previously described protocol [26]. Wells of 96-well plates were coated with 5 μg protein/50 μl of the DEAE extract diluted in PBS and the plate was incubated overnight at 4 C. Wells were washed three times with PBS and then incubated in blocking buffer (2 % bovine serum albumin [BSA; Sigma-Aldrich] in PBS) for two hours with minimal shaking. Wells were washed once with PBS and 100 μl of the appropriate concentration and conformation of Aβ1-42 or Aβ1-16 was added and the plates incubated at room temperature for 2 h. Wells were then washed three times with PBS and mouse anti-Aβ 4G8 (Covance; Dallas, TX) or mouse anti-Aβ 6E10 (Covance) diluted 1:2000 was added in blocking solution. After primary antibody addition, wells were washed three times with PBS and 100 μl of goat anti-mouse IgG conjugated to HRP (1:2000; Thermo Scientific, Rockford, IL) was added and the plate incubated for 1 h at room temperature. After washing, TMB substrate (Sigma-Aldrich) was added and wells were allowed to develop until the blank wells turned faint blue, and the reaction was stopped by adding 50 μl 1 M sulfuric acid. Absorption was read at 450 nm.

The same DEAE extract as described above was used for immunoprecipitation experiments. Briefly, 10 μg of DEAE extract was incubated with 2 μg of monomeric, oligomeric, or fibrillar Aβ1-42 in 1 ml of PBS containing 1 % BSA. Assuming that brevican is ~1/50^th^ of total DEAE, the concentration of brevican was ~1 nM with 444 nM Aβ1-42. The tubes were incubated at room temperature for 2 h, and then 5 μg of mouse anti-Aβ 4G8 (Covance) was added and incubation continued for 1 h. Washed and equilibrated Protein A Sepharose 4B beads (GE Healthcare Life Sciences) were added to the solution and incubated for 30 min with vortexing every 5 min. The beads were centrifuged, washed with 1 % BSA in PBS and 40 μl of 2x Laemmli sample buffer was added to the beads, incubated for 10 min with vortexing, centrifuged and supernatant recovered. For western blot, 30 μl of each sample was loaded onto 4-20 % SDS-PAGE gels, electrophoresed, transferred to PVDF membrane and probed with mouse anti-brevican (BD Biosciences 610895, San Jose, CA).

### Mice and surgery

Mouse protocols used in these experiments were approved by the Institute for Animal Care and Use Committee at the University of Arkansas for Medical Sciences and effort was made to limit the number of animals required in each experiment and any pain produced by the surgery. B6C3-Tg(APPswe,PSEN1dE9)85Dbo mice (APPswe/PS1dE9) were purchased from Jackson Laboratory (Bar Harbor, MN) and a live colony was maintained in our local vivarium by mating hemizygotes with nontransgenic animals. Litters from these matings were used for both non-transgenic controls and APPswe/PS1dE9 mice. Mice were aged to 14 months and then underwent surgery for brain injection of chondroitinase ABC (ChABC; Sigma Aldrich, St. Louis, MO). Mice were anesthetized with inhaled isoflurane and mounted on a Kopf stereotaxic apparatus (Tujunga, CA) with a specialized nose cone to accommodate brain surgery. The body was placed on a heating pad, the head sterilized and shaved, an incision made at the sagittal midline, and a hole was made in the skull using a high speed drill. A 1 μl injection of either ChABC (5 mU/μl), diluted in saline containing 0.1 % BSA, or saline with 0.1 % BSA was made using a 10 μl Hamilton syringe with a 32 gauge blunt end needle to the following site at the right dorsal hippocampus (distance in mm from bregma: posterior 1.8 mm, lateral 1.2 mm, ventral 1.2 mm). The injection was made over a two minute span and after the completion of the injection the needle was held in place for an additional two minutes. The needle was then removed, the hole closed with bone wax, the incision sutured, and the animal returned to a cage containing a heating pad for 30 min and then back to the regular cage. Eighteen days after surgery, mice were euthanized with pentobarbital and cardiac-aorta perfused with ice cold saline followed by 4 % paraformaldehyde. The brain was dissected and fixed overnight at 4 C in 4 % paraformaldehyde (Sigma-Aldrich). Prior to cryostat sectioning, the brains were incubated in 15 % and 30 % sucrose prepared in PBS. For detection of the length of time that ChABC reduces *Wisteria floribunda* agglutinin (WFA) staining (Fig. 3), four month old C57Bl/6 mice were injected with the same dose of ChABC and euthanized 1, 2 and 3 weeks following surgery, their brain collected and processed for histochemistry as described above. Sections were stained with WFA (Vector Laboratories, Burlingame, CA).

### Measurement of Aβ burden and synaptic density in stratum lacunosum moleculare (slm)

Free floating, 30 μm cryostat sections underwent immunofluorescence staining using standard protocols as described [24] with rabbit anti-Aβ 95-2-5; [24, 27–29], mouse anti-synaptophysin (MAB368; EMD Millipore) and mouse anti-PSD-95 (clone K28/74 Ab2315909; Neuromab, Davis, CA). Fluorescent secondary antibodies used were: goat anti-rabbit IgG conjugated to Alexa Fluor 594 (anti-Aβ) and goat anti-mouse IgG conjugated to Alexa Fluor 488 (anti-synaptophysin and anti-PSD-95). For each mouse, three sections were analyzed beginning with a random section where the lateral ventricles end at the ventral hippocampus, and the next two sections were each separated by 120 μm. Aβ burden was conducted on Aβ and PSD-95 stained sections and images were captured of the contralateral and ipsilateral (injected hemisphere) hippocampus at 100x magnification. Images were opened in Fiji Image J and the slm was outlined on the PSD-95 image. This outline was then transferred to the Aβ-stained image. Brightness, contrast and threshold was adjusted identically for all sections and the “area fraction” occupied by Aβ staining in the binary image was calculated. This was performed on both the contralateral and ipsilateral slm of ChABC injected sections (7 mouse brains, 3 sections each), as well as vehicle injected sections (3 mouse brains, 3 sections each).

Sections to measure synaptic density surrounding plaques in the slm were stained with anti-Aβ and anti-synaptophysin with the same secondary antibodies. Synaptophysin density was measured with similar distribution of sections. Plaques were identified in the slm and images were adjusted for brightness and contrast that was used in an identical manner for every section. The Aβ image was magnified to 150 % of the original; using the freehand selection tool on Image J, a freehand shape was drawn around the most intense region of the plaque. Once complete, the synaptophysin image was selected, and the “Restore Selection” command (Edit→Selection→Restore Selection) was used to move the freehand drawing onto the synaptophysin image in the identical location and density measured. The drawing was then moved to a similar non-plaque area in the un-injected hippocampus, density measured, and the density of the plaque was divided by the density of the non-plaque region. This procedure was then repeated for a region outside the intense region of the plaque and then a region that is ½ the width of the distance between the two previous freehand drawings. Thus, the y-axis term “Proportion of non-plaque density” in Fig. 5 refers to the ratio of synaptophysin density of the plaque region divided by the synaptophysin density of the non-plaque region. This proportion was calculated for the “inner”, “middle” and “outer” regions of the plaque.

### Statistical analysis

All analyses were performed using GraphPad Prism 5.0. *p* < 0.05 was considered a significant difference unless stated otherwise. Brevican density from human tissue on Western blots was evaluated by one way ANOVA followed by Bonferroni multiple comparison test. (NCI n = 14, MCI n = 10, AD n = 14). EC50s for the binding assays were determined with non-linear regression. The mean EC50s (n = 5 binding assays) for multiple conformational Aβ binding to DEAE purified brain protein was evaluated using one-way ANOVA followed by Tukey’s multiple comparison test. For Aβ burden the per cent area occupied and per cent ipilateral to per cent contralateral comparisons were performed using unpaired Student’s *t*-test (n = 7 per group). Evaluation of synaptophysin density was performed using one-way ANOVA followed by Tukey’s multiple comparison test. Significant difference between groups are listed as *p* < 0.05 or *p* < 0.01.

## Results

**Fig. 1:**
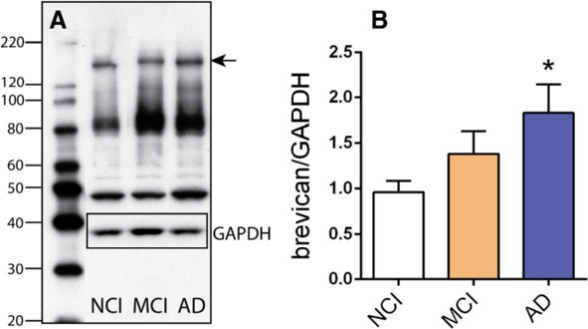
Western blot of human brevican from superior frontal gyrus of subjects with NCI (no cognitive impairment), MCI (mild cognitive impairment) and AD (Alzheimer’s disease). **a** Brevican blot of one lane from each group with intact brevican indicated by the arrow and GAPDH inset for normalization. **b** Quantification of intact brevican band density as a proportion of GAPDH density. Data were subject to one way ANOVA followed by Bonferroni multiple comparison test. Level of AD brevican is significantly elevated (*p* < 0.05) compared to NCI; (NCI n = 14, MCI n = 10, AD n = 14)

Increasing evidence has demonstrated that CS-bearing ECM molecules increase with age [20] and are associated with AD [19]. Thus, we assessed brevican content in superior frontal gyrus from no cognitive impairment (NCI), mild cognitive impairment (MCI) and Alzheimer’s disease (AD) subjects using immunoblot (for full blots, see Additional file 2: Figure S1). A clear elevation of intact, full-length brevican core protein content (Fig. 1a, arrow) was observed in AD superior frontal gyrus tissue compared to NCI individuals (Fig. 1a, b). Indeed, brevican in AD frontal gyrus was nearly double that of NCI tissue, and there was a clear trend of increased full length brevican in MCI subjects compared to NCI tissue.

**Fig. 2:**
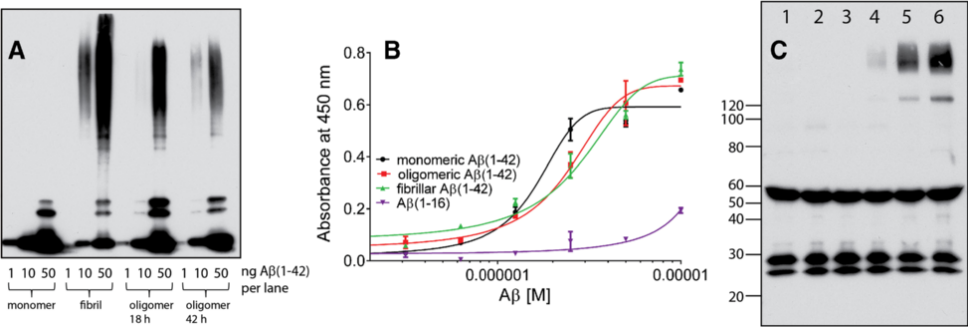
Binding of Aβ isoforms to DEAE purified molecules including brevican. **a**. Western blot of Aβ isoforms used in binding assay to DEAE products in “**b**”. **b**. Solid phase, dose-dependent, binding of Aβ1-42 isoforms (monomeric, oligomeric and fibrillar) and Aβ(1–16) to DEAE purified molecules. The mean EC50s (n = 5 binding assays) for multiple conformational Aβ binding to DEAE purified brain protein was evaluated using one way ANOVA followed by Tukey’s multiple comparison test. EC50 for monomeric, oligomeric and fibrillar Aβ1-42 is significantly lower compared to the EC50 for Aβ1-16; (*p* < 0.05). **c**. Immunoprecipitation of brevican with mouse anti-Aβ 4G8 after soluble binding to DEAE product. Western blot was performed with mouse anti-brevican antibody. Lane identities: lane 1 DEAE extract only; lane 2 fibrillar Aβ only; lane 3 4G8 antibody only; lane 4 DEAE extract + monomeric Aβ; lane 5 DEAE extract + oligomeric Aβ; lane 6 DEAE extract + fibrillar Aβ. Anti-Aβ 4G8 pulled down brevican bound to oligomeric (lane 5) and fibrillar Aβ (lane 6). Lower molecular weight bands correspond to heavy and light IgG chains. Although all three isoforms of Aβ1-42 bound to brevican, binding to monomeric Aβ was markedly lower than binding to oligomeric and fibrillar Aβ

Previous data has also shown that CS-bearing PGs bind to Aβ, but it is not known whether Aβ binds to brain-derived lecticans [10, 30]. Binding of Aβ to lecticans may enhance the inhibition of synaptic plasticity. Thus, an extract enriched with CS-bearing PGs was purified from whole mouse brain and this extract was used to perform a solid-phase binding assay with various forms of Aβ (Fig. 2a and b). We observed a dose-dependent increase in monomeric, oligomeric, and fibrillar Aβ1-42 binding to the DEAE extract (Fig. 2b). The EC50 for monomeric was 1.6 ± 0.12 μM, oligomeric was 2.4 ± 0.2 μM, and fibrillar was 2.7 ± 0.3 μM for variants of Aβ1-42 and 50.0 ± 3.0 μM for Aβ1-16. All three conformations of Aβ1-42 showed significantly higher affinity binding to CS-bearing PGs compared to Aβ1-16 (n = 5 assays; *p* < 0.05). Interestingly, when conducting soluble binding of various forms of Aβ1-42 with DEAE extract, immunoprecipitating with anti-Aβ antibody and probing for brevican on a Western blot, oligomeric and fibrillar Aβ showed greater binding to brevican compared to monomeric Aβ1-42 (Fig. 2c), although there was binding by all three forms.

**Fig. 3:**
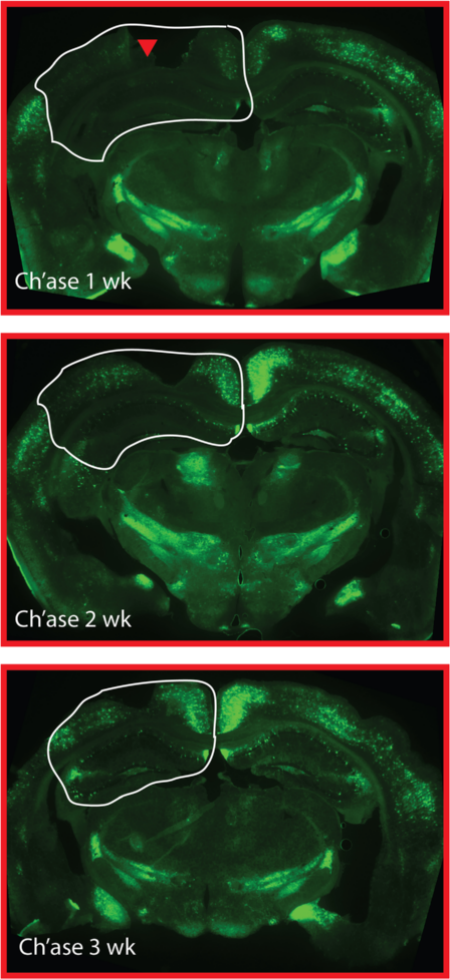
Intracerebral ChABC injection results in a marked and long-lasting decline in CS-bearing PGs. Low magnification micrographs (25x) of *Wisteria floribunda* agglutinin (WFA) staining 1, 2, and 3 weeks after ChABC injection (5 mU/1 μL). Arrowhead represents injection site for ChABC. Note the widespread loss of WFA immunoreactivity in the injected (left side) cortex and hippocampus (area with white outline). Injection target was to the ventral side of the corpus callosum on the dorsal side of dorsal hippocampus

Since it was demonstrated that Aβ and brain-derived lecticans do interact, it was next examined whether removal of CS chains from the lecticans would affect the presence of Aβ-containing plaques and the presence of a synaptic marker surrounding these Aβ plaques. Stereotaxically-positioned, APPswe/PS1dE9 and non-transgenic (nt) mice were injected with 5 mU of ChABC at 14 months of age at a location that was at the most dorsal hippocampus, on the ventral side of the corpus callosum. Previous studies have shown that a single injection of ChABC results in long-lasting removal of CS chains from a large region of the target area [31, 32]. Indeed, staining brain tissue from 3 months old mice that were previously injected with ChABC with *Wisteria floribunda* agglutinin (WFA, a lectin that detects carbohydrate structures that contain N-acetylgalactosamine and stains perineuronal nets [PNNSs] in brain) indicated a long-lasting decrease of WFA staining in the injected hemisphere (Fig. 3). A significant amount of cortex and a majority of the dorsal hippocampus showed a reduction in WFA of staining, an effect that persisted for at least 3 weeks following injection (Fig. 3). Thus, injection of ChABC into AD model tissue allows for evaluation of the effect of removal of CS on Aβ deposition into plaques and determination of this effect on synaptic density in regions surrounding these plaques.

**Fig. 4:**
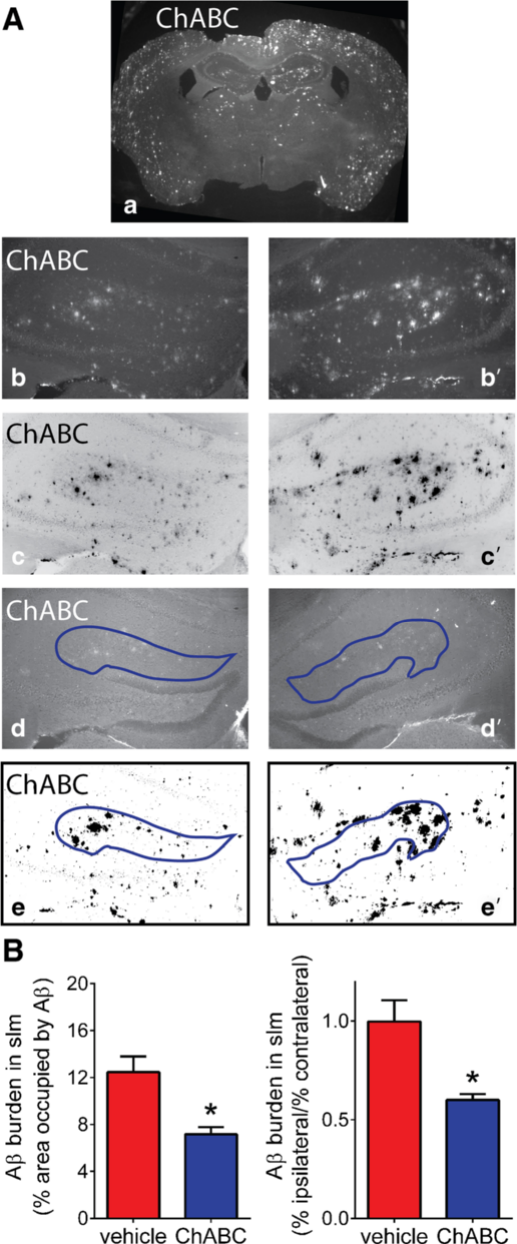
Injection of ChABC into APPswe/PS1dE9 dorsal hippocampus results in reduced amyloid burden in the stratum lacunosum moleculare (slm). **a** *a*. Immunostaining for Aβ (rabbit anti-Aβ 95-2-5) showing whole brain section of ChABC injected APPswe/PS1dE9 mouse hippocampus at low magnification (25x); **a** *b*,**a** *b’.* Aβ immunostaining of slm on ChABC injected ipsilateral (**a** *b*) and contralateral (**a** *b’*) sides (100x). **a** *c*,**a** *c’.* inverted images of (**a** *b* and **a** *b’*). **a** *d*,**a** *d’.* PSD-95 staining in the same sections as in (**a** *c*,**a** *c’*) with slm outlined in blue. **a** *e*,**a** *e’.* sections from (**a** *c*,**a** *c’*) thresholded and converted to binary to calculate the area occupied by Aβ. **b**. Quantitative measurement of area occupied by Aβ in the slm as area occupied by Aβ (left graph; APPswe/PS1dE9 n = 7) and area occupied as per cent of contralateral side (right graph, no ChABC treatment n = 3; ChABC treatment n = 7). Comparison between treatments was made using unpaired Student’s *t*-test. *p < 0.05 was considered a significant difference between groups

In APPswe/PSdE9 mice, 18 days after ChABC injection Aβ burden was measured in the stratum lacunosum moleculare (slm), one region of the hippocampus that contains numerous amyloid plaques. The slm was easily recognized when co-staining the sections for PSDS-95 (Figure 4a*d* and a*d’*). The reduction in Aβ plaques was observable in the ChABC-treated hemisphere (eg. Figure 4a*a*). The per cent of the slm occupied by Aβ after thresholding the immunoreactive density (Figure 4a*e*) was nearly 50 % lower in ChABC treated slm compared to the non-ChABC treated slm hemisphere (Fig. 4b, left graph; *p* < 0.05). When the ratio of ipsilateral/contralateral was calculated and compared to vehicle-treated APPswe/PS1dE9 mice, there was approximately a 40 % reduction in Aβ burden. Thus, removal of CS for 18 days after injection of ChABC resulted in a significant reduction in the presence of Aβ in the slm hippocampus in APPswe/PS1dE9 mice.

**Fig. 5:**
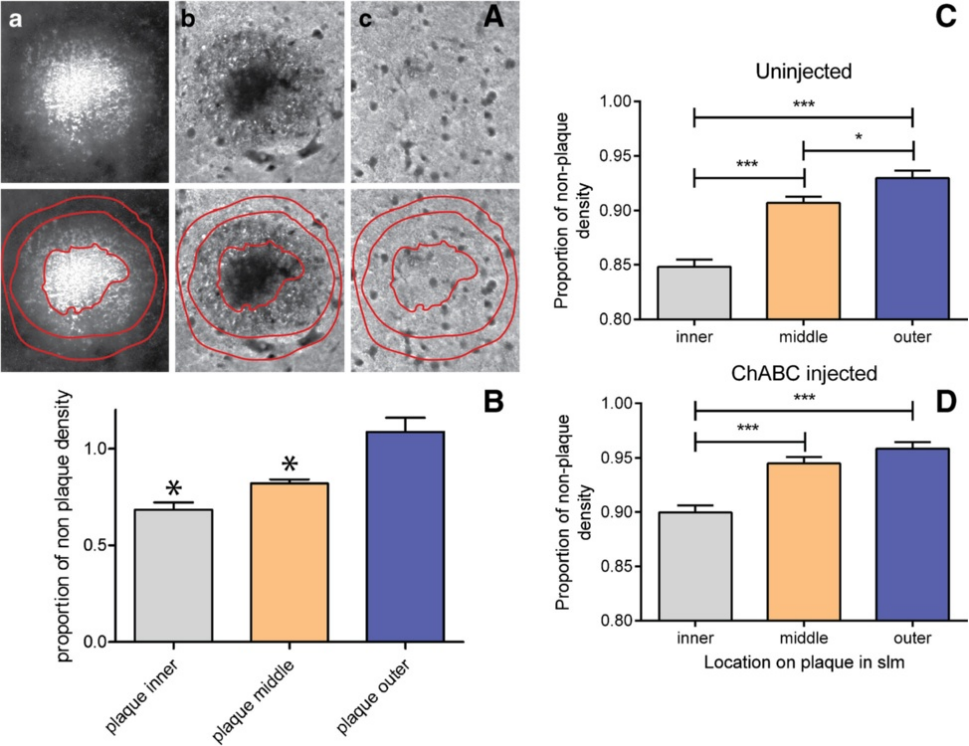
ChABC injection increases density of synaptophysin surrounding Aβ plaques in the slm. **a** *a.* freehand rings were drawn around three regions of an amyloid plaque (inner, middle and outer). **a** *b.* rings were transferred to the identical location on the synaptophysin image and density measured in each ring. **a** *c*. identically drawn rings were moved to an adjacent, non-plaque region and synaptophysin density measured. **b**. quantitative density measurements of synaptophysin immunoreactivity around plaques in slm of 15 months old APPswe/PS1dE9 mice. Measurements were made in the slm after ChABC injection on the (**c**) contralateral, non-injected hemisphere and the (**d**) injected, ipsilateral side. The y-axis term “Proportion of non-plaque density” refers to the ratio of synaptophysin density of the plaque region divided by the synaptophysin density of the non-plaque region. This proportion was calculated for the “inner”, “middle” and “outer” regions of the plaque. Note there was a significant loss of synaptophysin immunoreactivity between the outer and middle plaque layers on the contralateral (**c**) but not inpsilateral, ChABC-injected side (**d**). For (**b**) n = 5 mice, for (**c**) and (**d**) n = 7 mice, * *p* < 0.05; ***p < 0.001

Since ChABC significantly reduced Aβ plaque burden in the slm, it was next examined whether synaptic density surrounding the plaques was altered. Additional sections containing hippocampus from the same ChABC-treated mice were subjected to immunofluorescence for Aβ and synaptophysin. On the Aβ image, free hand rings were drawn around regions of the highest plaque density, the less dense “halo” area and then about 50 % of the distance from the first to the second ring (Figure 5a*a*). The rings were then moved to the synaptophysin stained image of the same section (Figure 5a*b*) and the density of each ring was determined. The rings were then moved to a “non-plaque” region adjacent to the plaque (Figure 5a*c*) and the density was measured and calculated on each ring to determine the plaque/non-plaque ratio. In an initial experiment prior to ChABC treatment, there was a significant decrease in synaptophysin density ratio in the middle and inner rings, compared to the outer ring (Fig. 5b). When the same experiment was conducted with ChABC injected animals, there was again a significant difference in synaptophysin density ratio between the middle and outer rings around plaques measured in the slm of the un-injected, contralateral hemisphere (Fig. 5c). However, there was no significant difference in the density of the middle and outer rings in the ChABC injected region (Fig. 5d). These data suggest that some mechanism related to the removal of CS chains was responsible for inducing synaptic plasticity that increased synaptophysin density in the middle region.

## Discussion

The results from this study have shown that intracerebral injection of ChABC into 15 months old APPswe/PS1dE9 mice increased synaptic density surrounding plaques and reduced Aβ burden in the slm of the hippocampus. Although the increase in synaptophysin density surrounding plaques in the slm of ChABC injected animals was quite modest, it was significantly different from the uninjected hemisphere. We demonstrated that Aβ binds to brain-derived lecticans, including brevican, molecules known to be involved in inhibition of synaptic plasticity. Since lecticans are elevated in AD frontal cortex brain tissue [19], this finding suggests that lecticans may play a role in diminishing synaptic plasticity in AD tissue. This role most likely involves brevican, since it is a lectican that is deposited as an extracellular, adjacent synaptic protein [6, 33]. Accordingly, the targeting of lecticans at the synapse could be a beneficial direction for eventual therapeutic development for AD.

The role of CS-bearing PGs in synaptic plasticity has been known for nearly two decades. It was originally demonstrated that alterations in deposition occurred in visual cortex following visual deprivation [34]. The ability of ChABC treatment to reinstate ocular dominance plasticity in a visually-deprived adult animal was the first result that implicated these PGs in nervous system plasticity [7]. More recent data has shown that PGs contribute to the stability of various learning and memory behaviors and that ChABC treatment can enhance plasticity for recall [9, 35]. In addition, levels of hippocampal brevican and versican were increased in rats exposed to training in the Morris water maze, and immunoprecipitation of brevican and versican demonstrated that these lecticans bind to the GluR1 subunit of the AMPA receptor [36]. Thus, the major loss of synaptic plasticity in progressive AD is presumed to be due to the pathological functions of Aβ and tau [37]; however, it is possible that CS-bearing PGs may have some input into the process as well. There have been two prior studies that have examined how treatment with ChABC can induce plasticity in brain of AD model mice. One study demonstrated that young, 3 months old APPswe/PS1dE9 mice that had not developed plaques had elevated matrix protein levels, an impaired ability to form contextual fear memory, and a decline in hippocampal long term potentiation [21]. Hippocampal injection of ChABC reinstated both fear memory behavior and long term potentiation [21]. Interestingly, matrix proteins were not significantly elevated in 6 or 12 months old APPswe/PS1dE9 mice. This finding suggests that elevated lecticans may play a role in the early memory loss in this model. Data from the current study adds to this hypothesis, where even in aged APPswe/PS1dE9 mice, ChABC treatment induced synaptic plasticity in regions that surround plaques in the slm of the hippocampus. These regions surrounding plaques have been shown to contain elevated levels of soluble oligomeric Aβ that associates with post-synaptic densities [38]. It would be interesting to determine whether there is an interaction of Aβ with CS-bearing PGs such as brevican in these adjacent synaptic regions that are near senile plaques. In two different mouse models of tau pathology, although ChABC treatment did not affect the progression of the neurodegenerative disease state, it did restore plasticity by reversing the decline in synaptic transmission and improved object recognition memory in both models [39]. Although classically tau has been thought of as an axonal microtubule-related protein, more recent data indicates that it is more widely distributed in neurons and in AD tissue, a truncated fragment of tau may be secreted from pre-synaptic terminals [18]. Thus, ChABC may be exerting its action at the synapse in the tauopathy models as well.

Unique to this study is the observation that over time, the removal of CS chains in the CNS by intracerebral administration of ChABC reduces Aβ bearing plaques and plaque size in APPswe/PS1dE9 mice hippocampus. It is known that the formation of Aβ42 bearing fibrils is enhanced in the presence of polymeric glycosaminoglycans [40] and most importantly, the binding of Aβ42 to glycosaminoglycans increased the resistance of Aβ42 to proteolysis 2 to 5-fold; these findings suggested that elimination of CS chains in AD models brain should reduce plaques by enhancing the degradation of Aβ42 [26, 40]. The present data supports this hypothesis in a heavy plaque-bearing region of the hippocampus, the slm, in ChABC-treated APPswe/PS1dE9 mice. In fact, a single intra-hippocampal injection reduced Aβ load by almost 50 % 18 days after injection. Thus, ChABC injection may be beneficial in stimulating plasticity, as well as reducing Aβ burden.

Substantial supporting evidence suggests that not only can CS-bearing PGs exert negative actions on plasticity mechanisms in models of neurodegenerative disease, but that other areas of expression of these agents may be quite beneficial. Significant evidence exists that suggests that aggregates of matrix lattice that include lecticans, hyaluronic acid and tenascin-R, that exist as PNNs are protective toward neuronal cell bodies. Indeed, under several neurodegenerative conditions *in vivo* and *in vitro*, the presence of CS-bearing PGs and/or PNNs have been shown to be neuroprotective [30, 41–44]. In AD brain, neurons in cortical regions bearing matrix PNNs were less affected by neurofibrillary tangle pathology [3]. Indeed, tau pathology was located in CNS regions with a low proportion of neurons with PNNs, and overall no changes were observed in numbers or structure of PNNs [45, 46, 47]. One study implicated that increases in brevican and cartilage link protein in AD may exist in functional peri-synaptic regions, a hypothesis requiring further support [19]. These data provide the insight that not only do PNNs potentially protect neurons from cell death in AD, but that perisynaptic matrix maintains synapse integrity in neurodegenerative disease. The present findings most likely support the notion that the presence of perisynaptic matrix contributes to the inhibition of synaptic plasticity in AD, and that removal of CS chains from this matrix would stimulate plasticity.

Future studies may focus on defining the mechanism of CS-bearing PGs involvement in neuroprotection and synaptic plasticity. Most of these studies have been conducted with differential means of altering the expression or structure of CS-bearing PGs. Although it is evident that CS on these core proteins is clearly important in inhibition of synaptic plasticity, the contribution of CS or the larger matrix aggregate in the neuroprotective properties remains to be distinguished.

## Conclusions

Hippocampal injection of ChABC in the APPswe/PS1dE9 mouse model of AD resulted in reduced amyloid burden and increased synaptic marker density surrounding plaques in the slm of the hippocampus. This suggests that because brevican binds to Aβ, the reduction in Aβ and loss of CS chains from brevican located in synaptosomal regions may enhance synaptic plasticity. Enhancing synaptic plasticity in early AD, either with ChABC treatment or by reductions in brevican signaling may be a significant target that could slow disease progression.

## Additional files

Additional file 1: Table S1. Profile characteristics of subjects whose superior frontal gyrus tissue was measured for brevican. (PDF 121 kb) Additional file 2: Figure S1. Full Western blot of extracted samples from human superior frontal cortex obtained from subjects diagnosed as NCI (no cognitive impairment), MCI (mild cognitive impairment), and AD (Alzheimer’s disease). Samples were measured using four separate gels, each with a “mixed” internal control (IC) sample. The top arrow indicates the band of full length brevican in these tissues, and the bottom arrow points to the bands of GAPDH for these samples. Samples 5927NCI, 3499MCI and 9365AD were eliminated from the calculation due to their being outliers for this analysis. Methods for performing the western blot and analyzing the data are inclusing in the “Materials and Methods” section of the regular manuscript. (JPEG 1407 kb)

## Abbreviations

Aβ: amyloid-β
AD: Alzheimer’s disease
APP: amyloid precursor protein
BSA: bovine serum albumin
ChABC: chondroitinase ABC
CS: chondroitin sulfate
DEAE: diethylethanolamine
DMSO: dimethylsulfoxide
ECM: extracellular matrix
HFIP: hexafluoro-2-propanol
HRP: horseradish peroxidase
MCI: mild cognitive impairment
NCI: no cognitive impairment
PBS: phosphate buffered saline
PG: proteoglycan
PS1: presenilin-1
WFA: *Wisteria floribunda* agglutinin
