# Supplementary material for: Hippocampal administration of chondroitinase ABC increases plaque-adjacent synaptic marker and diminishes amyloid burden in aged APPswe/PS1dE9 mice
Source: Acta Neuropathol Commun. 2015 Sep 4;3:54. doi: 10.1186/s40478-015-0233-z (PMC4559967; doi:10.1186/s40478-015-0233-z)
Supplement: Additional file 1: Table S1. — Profile characteristics of subjects whose superior frontal gyrus tissue was measured for brevican. (PDF 121 kb) [file 40478_2015_233_MOESM1_ESM.pdf]

ESM Table 1. Profile characteristics of subjects whose superior frontal gyrus tissue was measured for brevican. Abbreviations and descriptions of abbreviations: Cogn Diagn – cognitive diagnostic category; PMI – post-mortem interval in hours; apoE alleles – apolipoprotein E alleles; MMSE – mini mental state examination score; int of MMSE – interval between last MMSE and death; Braak SC – Braak score (0=0, 1=I, 2=II, 3=III, 4=IV, 5=V); Cerad AD – CERAD diagnosis (4=no AD, 3=possible AD, 2=probable AD, 1=definite AD); Reagan AD – NIA/Reagan diagnosis (4=no AD, 3=low likelihood, 2=intermediate likelihood, 1 high likelihood); NCI – no cognitive impairment; MCI – mild cognitive impairement; AD – Alzheimer's disease

Case ID no	Cogn Diagn	Age Death	Gender	PMI (h:min)	apoE alleles	latest MMSE	int of MMSE days	Braak SC	Cerad AD	Reagan AD
10203224	NCI	83.3	M	7:15	E3E3	'30/30'	356	0	2	3
21001933	NCI	80.9	F	4:50	E3E3	'27/30'	42	1	3	3
20875195	NCI	80.9	F	4:05	E3E3	'28/30'	595	2	4	3
20942767	NCI	77.3	F	16:15	E3E3	'30/30'	54	4	3	3
15115927	NCI	77.4	M	10:05	E3E3	'30/30'	190	1	4	3
20956867	NCI	79.2	F	18.13	E3E3	'30/30'	125	3	4	3
20665307	NCI	95.3	F	5:20	E3E3	'30/30'	190	3	2	2
20270920	NCI	72.7	F	14:58	E2E3	'27/30'	39	3	4	4
10490993	NCI	86.6	M	7:00	E3E3	'27/30'	342	5	2	2
20993308	NCI	83.4	F	1:00	E3E3	'26/30'	62	1	4	3
20521752	NCI	78.2	F	8:04	E3E3	'30/30'	129	3	3	3
11072071	NCI	88.0	M	85:05	E3E3	'27/30'	356	2	4	3
11259716	NCI	92.6	M	6:00	E3E3	'28/30'	58	3	2	2
10606903	NCI	80.4	M	26:30	E3E4	'23/30'	354	3	2	2
10253148	NCI	89.9	M	21.04	E3E3	'20/22'	32	5	2	2
10439580	MCI	91.4	M	3:30	E3E3	'24/30'	0	3	4	3
21405627	MCI	94.1	F	1:50	E3E3	'28/30'	115	3	4	3
20139850	MCI	86.4	F	3:50	E3E3	'27/30'	27	4	4	3
10288185	MCI	76.9	M	3:15	E4E4	'20/22'	340	3	4	3
10101291	MCI	83.5	M	7:35	E2E3	'28/30'	335	3	4	3
15773499	MCI	89.3	M	13.25	E3E4	'24/30'	38	4	1	2
21232244	MCI	97.6	F	9:55	E3E3	'25/30'	253	4	2	2
20154287	MCI	90.1	F	2:45	E2E3	'26/30'	350	3	4	3
20974481	MCI	96.7	F	15:44	E3E3	'26/30'	292	4	2	2
21402016	MCI	91.8	F	1:45	E3E4	'28/30'	29	5	1	1
10249336	MCI	75.0	M	16:00	E3E4	'27/30'	251	3	2	2
21412752	AD	99.3	F	1:57	E3E3	'23/30'	318	4	2	2
20611993	AD	89.7	F	11:15	E3E3	'3/30'	282	5	2	1
20970441	AD	96.2	F	13:30	E2E3	'19/30'	359	5	1	1
20922259	AD	98.5	F	4:40	E3E3	'2/30'	55	3	2	2
20953832	AD	81.8	F	5:20	E2E3	'20/30'	88	2	3	3
20978133	AD	87.7	F	26:10	E3E3	'21/30'	267	5	1	1
20929774	AD	88.0	F	23:20	E3E3	'22/30'	327	4	2	2
20945666	AD	99.2	F	17.25	E3E3	'22/30'	134	4	2	2
22101004	AD	85.8	F	23:00	E3E4	'5/30'	344	5	1	1
15179365	AD	83.7	M	3:00	E3E4	'17/30'	440	3	1	2
15420223	AD	85.4	M	7:25	E3E3	'16/30'	153	1	4	3
11453772	AD	80.4	M	1:07	E2E3	'14/30'	43	2	3	3
20630946	AD	104.3	F	2:54	E3E3	'0/30'	126	3	4	3
15121461	AD	93.5	M	5:50	E3E4	'16/30'	384	5	1	1
20603141	AD	91.3	F	5:00	E2E3	'25/30'	194	3	3	3
