# Supplementary figures and images for: Hippocampal administration of chondroitinase ABC increases plaque-adjacent synaptic marker and diminishes amyloid burden in aged APPswe/PS1dE9 mice
Source: Acta Neuropathol Commun. 2015 Sep 4;3:54. doi: 10.1186/s40478-015-0233-z (PMC4559967; doi:10.1186/s40478-015-0233-z)

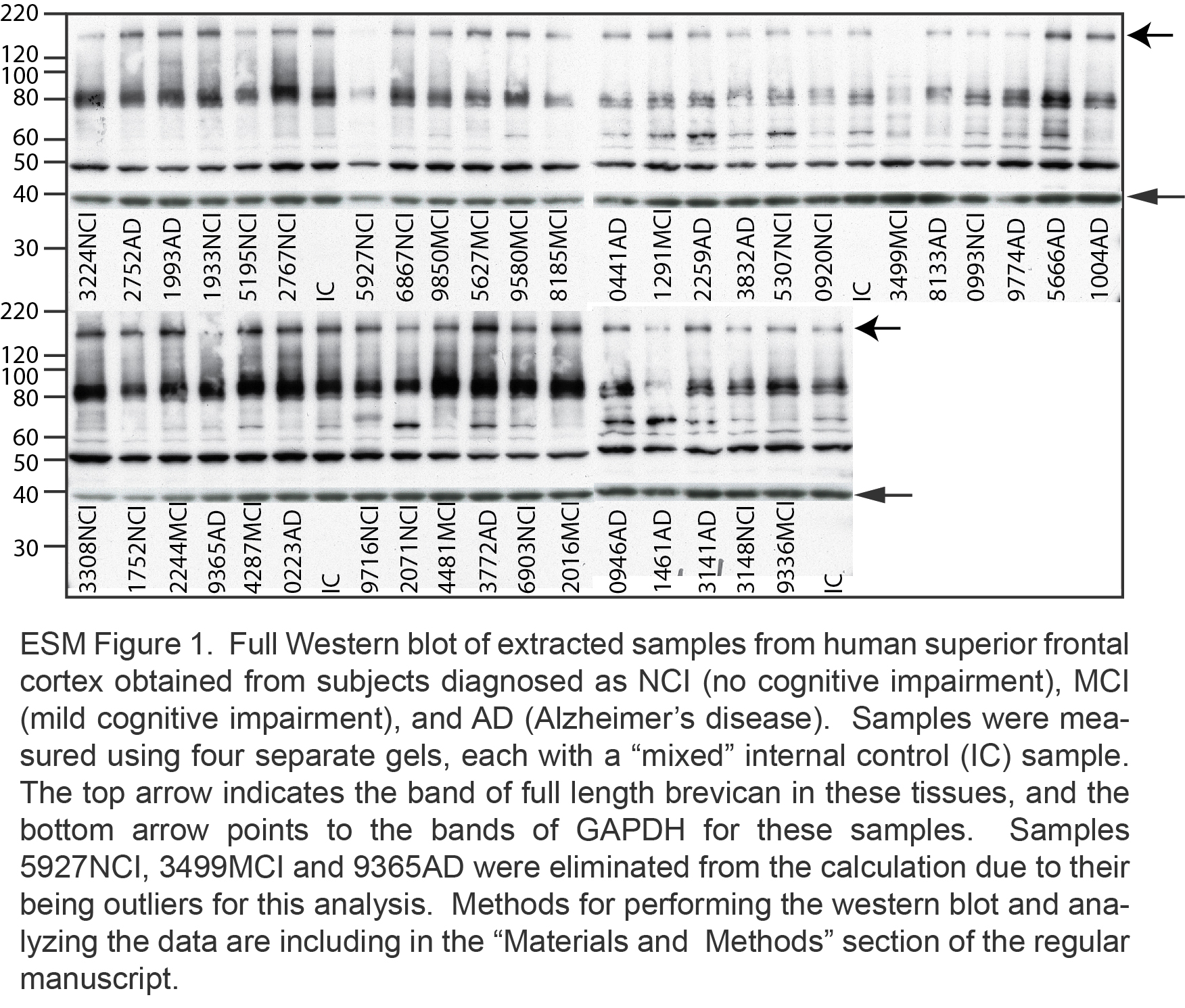

Supplement: Additional file 2: Figure S1. — Full Western blot of extracted samples from human superior frontal cortex obtained from subjects diagnosed as NCI (no cognitive impairment), MCI (mild cognitive impairment), and AD (Alzheimer’s disease). Samples were measured using four separate gels, each with a “mixed” internal control (IC) sample. The top arrow indicates the band of full length brevican in these tissues, and the bottom arrow points to the bands of GAPDH for these samples. Samples 5927NCI, 3499MCI and 9365AD were eliminated from the calculation due to their being outliers for this analysis. Methods for performing the western blot and analyzing the data are inclusing in the “Materials and Methods” section of the regular manuscript. (JPEG 1407 kb) [file 40478_2015_233_MOESM2_ESM.jpg]
